# Value of biomarkers in the prediction of shunt responsivity in patients with normal pressure hydrocephalus

**DOI:** 10.1007/s10143-025-03581-3

**Published:** 2025-06-03

**Authors:** Miroslav Cihlo, Pavel Trávníček, Alena Tichá, Radomír Hyšpler, Marta Kalousová, Svatopluk Řehák, Karel Zadrobílek, Lucie Kukrálová, Pavel Póczoš, Jan Pospíšil, Pavel Dostál, Vlasta Dostálová

**Affiliations:** 1 Department of Neurosurgery, Charles University, Faculty of Medicine in Hradec Kralove, Hradec Kralove, Czech Republic; 2 Department of Neurosurgery, University Hospital Hradec Kralove, Hradec Kralove, Czech Republic; 3 Department of Clinical Biochemistry, University Hospital Hradec Kralove, Hradec Kralove, Czech Republic; 4 Institute of Medical Biochemistry and Laboratory Diagnostics, First Faculty of Medicine, Charles University and General University Hospital in Prague, Prague, Czech Republic; 5 Department of Anaesthesiology and Intensive Care Medicine, Charles University, Faculty of Medicine in Hradec Kralove, Hradec Kralove, Czech Republic; 6 Department of Anaesthesiology and Intensive Care Medicine, University Hospital Hradec Kralove, Hradec Kralove, Czech Republic

**Keywords:** Normal pressure hydrocephalus, Biomarkers, NfL, NfH, NSE, S100B, Tau protein, Beta-amyloid

## Abstract

Preoperative differentiation between responders and non-responders to ventriculoperitoneal (VP) shunting in the treatment of normal pressure hydrocephalus (NPH) remains a significant challenge. Identifying biomarkers in presurgical assessment represents a promising approach to reducing the need for invasive cerebrospinal fluid CSF testing. In this prospective observational study, thirty adult patients were classified into Group A (responders to VP shunting) and Group B (non-responders) based on their responsiveness to invasive CSF testing. The overall clinical condition and Idiopathic NPH (iNPH) scale were assessed at baseline. Additionally, biomarker levels were compared between the two groups. Elevated levels of Neurofilament Light Chain (NfL) and Neurofilament Heavy Chain (NfH) in CSF and a reduced level of beta-amyloid Aβ42 were observed. No significant differences in biomarker levels were found between groups. Individual biomarkers demonstrated only poor predictive value (AUC = 0.37–0.53). Clinical factors were stronger predictors (AUC = 0.642–0.669), with no improvement when combined with all examined biomarkers (AUC = 0.428–0.431). No single biomarker reliably predicted confirmed postoperative shunt responsiveness among patients who underwent VP shunt placement and demonstrated clinical improvement. Clinical factors were stronger predictors, suggesting that patient history and clinical assessment (e.g., the iNPH scale) provide more reliable diagnostic information. Notably, combining biomarkers with clinical factors did not improve predictive accuracy.

## Introduction

Normal pressure hydrocephalus (NPH) is a neurodegenerative disorder of unclear etiology, characterized by Hakim’s triad: cognitive impairment (dementia), gait disturbances, and urinary incontinence [1–4]. These symptoms, which frequently occur in the elderly population, often overlap with the symptoms of"other"neurodegenerative diseases, especially Alzheimer’s disease and other (pre)senile dementias. Distinguishing NPH from"other"dementias is crucial in determining whether a patient could benefit from a surgical procedure—ventriculoperitoneal (VP) shunt placement or not [5–12]. Extensive evaluation of responsiveness to VP shunting, which consisted of a stepwise cerebrospinal fluid diagnostic assessment including tap test (TT), lumbar infusion test (LIT), and evaluation of the effects of external lumbar drainage placement (ELD) is currently considered a gold standard. This procedure is highly invasive, time consuming and caries a risk of meningitis. Search for other less invasive approaches to predict patient responsiveness to ventriculoperitoneal shunt placement is therefore of a high clinical interest. Elevated levels of biochemical markers of neuronal damage have been associated with various central nervous system pathologies. The most studied markers include Neurofilament Light Chain (NfL), Neuron-Specific Enolase (NSE), S100 protein, and tau protein, beta- amyloid Aβ1–40, Aβ42, GFAP and others [13–15].

### Neurofilament Light Chain (NfL) and Neurofilament Heavy Chain (NfH)

Neurofilaments (Nfs) are cylindrical proteins found exclusively in neuronal cytoplasm. Elevated levels of Nfs have been observed in response to axonal damage in the central nervous system (CNS) due to inflammatory, neurodegenerative, traumatic, or vascular causes, especially in older individuals. The concentration of NfL in cerebrospinal fluid is higher in patients with neurological disorders compared to healthy controls. Similar findings have been reported regarding NfL levels in serum. The role of NfL as a biomarker has been established in conditions such as multiple sclerosis (MS), Alzheimer’s disease (AD), frontotemporal dementia (FTD), amyotrophic lateral sclerosis (ALS), atypical parkinsonian disorders (APD), and traumatic brain injury (TBI). There is a stronger correlation observed between NfL levels and ALS compared to NfH, although the role of NfH has not been as extensively established yet.

### Protein S100

S100 protein belongs to a large family of calcium-binding proteins and is a well-established biomarker in many areas of medicine. S100B protein is a recognized biomarker in neurodegenerative diseases. Several studies have shown a significant elevation in the levels of S100B in cases of hydrocephalus. However, the relationship between S100B levels and clinical course is not sufficiently explored [16, 17].

### Neuron-Specific Enolase (NSE)

NSE is an isoenzyme in the glycolytic pathway. Increased levels of NSE have been described in patients with brain injury or stroke. Its half-life in serum is approximately 48 h [18–20]. Elevated levels of both S100B and NSE after injuries are associated with an unfavourable outcome and a greater extent of brain tissue damage. In a recent study by Mehmedika-Suljić et al., “*a non-significantly lower level of NSE was found in patients with hydrocephalus compared to healthy controls”* [21]. There is a limited number of studies focused on the significance of NSE and its clinical relevance in NPH.

### Tau Protein and Beta-Amyloid

There is a close overlap between idiopathic normal pressure hydrocephalus (iNPH) and Alzheimer’s disease (AD) because both conditions involve abnormal deposition of toxic byproducts of cerebral metabolism in the brain, such as amyloid-beta 1–42 (amyloid-β 1–42) and Tau protein. Evidence from brain tissue studies even suggests that iNPH could be considered a disease model for Alzheimer’s disease. It has also been demonstrated that Alzheimer’s disease is a strong predictor of non-responsive iNPH. Recent studies even propose that the final step in neurodegeneration is the pathological cerebral aggregation of toxic brain metabolism byproducts caused by disrupted clearance of these waste products, such as the deposition of amyloid-β 1–42 and Tau in Alzheimer’s disease and α-synuclein in Parkinson’s disease [22, 23].

Although biomarkers have been proposed as potential predictors of VP shunt responsiveness, their clinical utility remains uncertain. Previous studies have yielded inconsistent results, and no biomarker has been validated for routine clinical use. This study aims to systematically assess the predictive performance of selected biomarkers and compare their diagnostic value with established clinical factors.

Specifically, this study examines differences in biomarker levels between responders and non-responders, as identified by invasive CSF testing, and evaluates their potential utility in predicting shunt responsiveness relative to clinical assessment. The selection of biomarkers in this study was based on prior evidence of their association with neuronal injury and hydrocephalus-related pathology. NfL and NfH are established markers of axonal damage. Tau and beta-amyloid are linked to Alzheimer’s disease pathology, which often overlaps with iNPH. NSE and S100B have been investigated in acute and chronic brain injury and may reflect glial and neuronal stress responses [13–31].

## Materials and methods

This single-center, prospective observational cohort study was approved by the ethical committee (approval no: 202311 P02) of the University Hospital Hradec Kralove, Hradec Kralove, Czech Republic (Chairperson Jiri Vortel, MD) on December 10, 2023. This trial has been registered at ClinicalTrials.gov (NCT06083233).

Participants if this study were recruited between January 2024 and September 2024. Potential participants were identified based on the neurologist’s recommendation and after evaluation in our specialized outpatient clinic. All patients provided written informed consent for participation. Inclusion criteria were age > 50 years, diagnosed communicating hydrocephalus on MR or CT, MMSE score > 10 points, and absence of any structural lesion on MRI or CT. Exclusion criteria included known non-communicating hydrocephalus, structural lesion on MRI or CT (tumour, contusion, aneurysm), MMSE ≤ 10 points, life-expectancy shorter than 1 year, pre-existing other type of dementia (m. Alzheimer, vascular dementia). All participants underwent a basic evaluation of responsiveness to VP shunting, which consisted of a stepwise cerebrospinal fluid diagnostic assessment including tap test (TT), lumbar infusion test (LIT), and external lumbar draingage placement (ELD). The patient’s gait and balance were monitored using a gait test (shortened to 5 m for our purposes, where the number of steps, 5 m of walking time, and step length were evaluated), along with the MMSE score and the evolution of incontinence. Idiopathic Normal Pressure Hydrocephalus scale (INPH) was calculated and evaluated before the intial lumbar puncture and 4 h afterward. In the case of TT, gait and MMSE were assessed 24 h post-test, and ELD was evaluated 3 days after placement. In case of a positive response to shunt-responsivity testing (primarily assessed based on gait parameters and MMSE), surgical treatment (ventriculoperitoneal shunt placement) was indicated. This process divided the patients into Group A (anticipated responders to VP shunting), who underwent insertion of VP shunt and Group B (anticipated non-responders) who did not undergo any further treatment. All patients in Group A subsequently underwent ventriculoperitoneal (VP) shunt surgery and were clinically evaluated at 3 months postoperatively using the iNPH scale. The change in iNPH score served as an objective criterion to confirm postoperative clinical improvement and validate Group A as true responders.

The CSF samples were examined by ELISA method. The concentration of NfH was measured by Human phosphorylated Neurofilament H (BioVendor Group, Brno, Czech Republic), concentration of NfL in CSF by Neurofilament light for CSF samples (Uman diagnostics, Umea, Sweden), tau protein by Human Tau proteins (Cusabio, Houston, TX, USA), the concentration of NSE by Human neuron-specific enolase (cusabio, Houston, TX, USA), the concentration of S100B by Human S100B (BioVendor Group, Brno, Czech Republic) and the concentration of beta-amyloid Aβ42 by Human Ab42 Ultrasensitive kit (Thermon Fisher ScientificVienny, Austria). The concentration of NfL in serum was measured by SIMOA method.

The results were statistically processed using JASP (Eric-Jan Wagenmakers, room G 0.29, Department of Psychological Methods, University of Amsterdam, Nieuwe Achtergracht 129B, Amsterdam, The Netherlands, https://jasp-stats.org/), Google Colab (www.colab.google.com, USA), Microsoft Excel 365 (Microsoft, USA, www.office.com) have been employed to making graphs and descriptive statics. A power analysis based on an α error of 0.15 and β error of 0.1 was performed using JASP. A difference of 80% in each marker’s level was considered significant for the power analysis. The sample size required for the t-test (difference between two dependent means-matched pairs) was calculated to be 28 patients. A sample size of 36 patients was considered to compensate for potential dropouts and possible inaccuracies in the power analysis.

Missing data were minimal (< 5%) and handled using pairwise deletion to preserve statistical power while minimizing bias. Logistic regression was employed due to its interpretability and suitability for identifying the predictive value of individual variables. To capture potential non- linear relationships and interactions, Random Forest modeling was also applied. Cross- validation was used to assess generalizability and reduce overfitting.

The homogeneity of variances between the two groups during the analysis was evaluated using Levene’s test. The chi-square test was utilized to assess differences in postoperative gait – improvement. Mann–Whitney U Test was employed to compare differences in the INPH scale between groups and in the levels of biomarkers. Descriptive Statistics was employed to calculate means, medians, standard deviations, interquartile range. ROC analysis, cross- validation and Random Forest model were employed for assessment of predictive value with calculation of AUC.

## Results

**Fig. 1:**
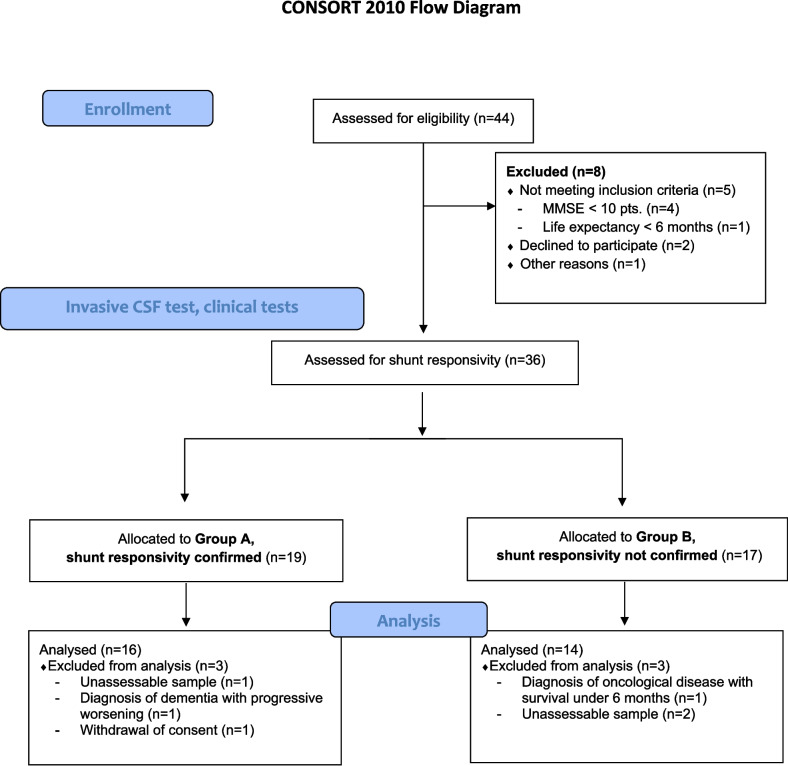
Consolidated Standards of Reporting Trials (CONSORT) 2010 flow diagram

A total of 30 patients were included in the study based on the fulfilment of inclusion criteria (Fig. 1). Sixteen patients were assigned to Group A (VP shunt responders) and fourteen to Group B (VP shunt non-responders). The baseline dataset is summarized in Table 1. There were no differences in baseline characteristics between groups.

**Table 1:** The baseline demographic characteristic, comorbidities, and results of functional tests

	Group A	Group B	*P*-value
	(*n* = 16)	(*n* = 14)	
Age (years) – median [IQR]	72.7 [69.5–79.0]	73.7 [68.5–76.3]	0.572
Sex (males/females)	9/7	11/3	0.190
MMSE (pts.) median [IQR]	25.5 [23.7–29.2]	22.9 [19.5–27.5]	0.168
Gait (steps)^1^	15.7 ± 9.1	16.2 ± 8.8	0.810
Time (sec.)^2^	10.75 ± 4.05	11.27 ± 4.34	0.768
Step length (m)^3^	0.23 ± 0.16	0.25 ± 0.18	0.715
Incontinence	8 (50.0%)	5 (35.7%)	0.435
INPH^4^ median [Q_1_-Q_3_]	60.5 [43.6 − 75.0]	48.0 [45.5 − 65.2]	0.053
Diabetes mellitus	31.2%	14.3%	0.274
Arterial hypertension	68.7%	78.6%	0.546
Ischemic stroke history	6.3%	0%	1.000
History of carcinoma	0%	0%	1.000
Ischemic heart disease, ischemic lower limb disease	12.6%	7.1%	0.565
Other causes of dementia	0%	0%	1.000

*p < 0.05 vs baseline value; ^1^The number of steps in the shortened 5-m walking test; ^2^Time in the shortened 5-m walking test; ^3^Calculated as the number of steps per 5 m; ^4^INPH = Idiopathic Normal Pressure Hydrocephalus scale

**Fig. 2:**
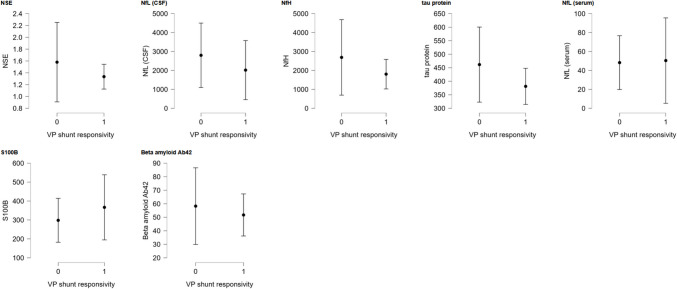
Plot graphs showing levels of each biomarker and normal level for patients. NSE = Neuron-Specific Enolase; NfL = Neurofilament Light Chain; NfH = Neurofilament Heavy Chain; CSF = cerebrospinal fluid; VP shunt reponsivity 0 = non-responders; VP shunt reponsivity 1 = responders

An elevation was observed in NfL in CSF and NfH, while a lower-than-normal levels were found in beta-amyloid Aβ42 CSF levels. As shown in Table 2 and Fig. 2, no differences in levels of all examined biomarkers between groups (*p* < 0.05) were observed. The highest ratio between groups was found for NfL in CSF (1.75), but, interestingly, this without any significance. Interestingly, the tau protein ratio was 0.91, indicating a higher level in Group A. Also, no difference was observed in the NfL CSF/serum ratio between groups (42.28 [34.16—82.39] in Group A vs. 54.16 [26.52—115.24] in Group B, p = 0.497). We also explored potential correlations between biomarker levels and patient age or symptom duration. No associations were observed (all *p* > 0.05, Spearman’s correlation).

**Table 2:** Baseline comparison of biomarkers associated with neuronal damage. Baseline cerebrospinal fluid and serum levels of biomarkers associated with neuronal damage in patients classified as responders (Group A) and non-responders (Group B). Values are presented as median [interquartile range]. No statistically significant differences were observed between groups

	Group A (*n* = 16)	Group B (*n* = 14)	Normal range	Ratio (B/A)	*P*-value
NSE^1^ (CSF^#^) [ng/ml]	1.28 [1.07—1.64]	1.35 [0.93—1.57]	< 2	1.05	0.984
NfL^2^ (CSF^#^) [pg/ml]	**892.60 [488.67–1473.24]**	**1569.06 [780.78–3448.92]**	< 600	1.75	0.240
NfH^3^ (CSF^#^) [pg/ml]	**1180.93 [575.93–2917.06]**	**1146.90 [393.96–2652.61]**	< 200	0.97	0.790
tau-protein (CSF^#^) [pg/ml]	411.75 [230.72–492.88]	381.75 [297.33–493.05]	< 500	0.93	0.822
NfL^2^ (serum) [pg/ml]	21.05 [10.43—31.87]	25.53 [13.31—60.24]	20–30	1.21	0.511
S100B [pg/ml]	212.46 [142.42–449.14]	240.70 [165.22–352.67]	2000–5000	1.13	1.000
Beta-amyloid Aβ42 [pg/ml]	**51.13 [30.44–60.48]**	**46.44 [28.71–69.91]**	≥ 550	0.91	0.803

*p < 0.05 vs baseline value; ^1^NSE = Neuron-Specific Enolase; ^2^NfL = Neurofilament Light Chain; ^3^NfH = Neurofilament Heavy Chain; ^#^CSF = cerebrospinal fluid

All patients in Group A underwent VP shunt surgery and completed a postoperative follow- up at 3 months. A significant improvement was observed in their clinical status as measured by the INPH scale. The median INPH score increased from 60.5 [47.4–77.6] preoperatively to 77.4 [69.6–91.6], p = 0.00003. This confirms that Group A consisted of true postoperative responders.

**Fig. 3:**
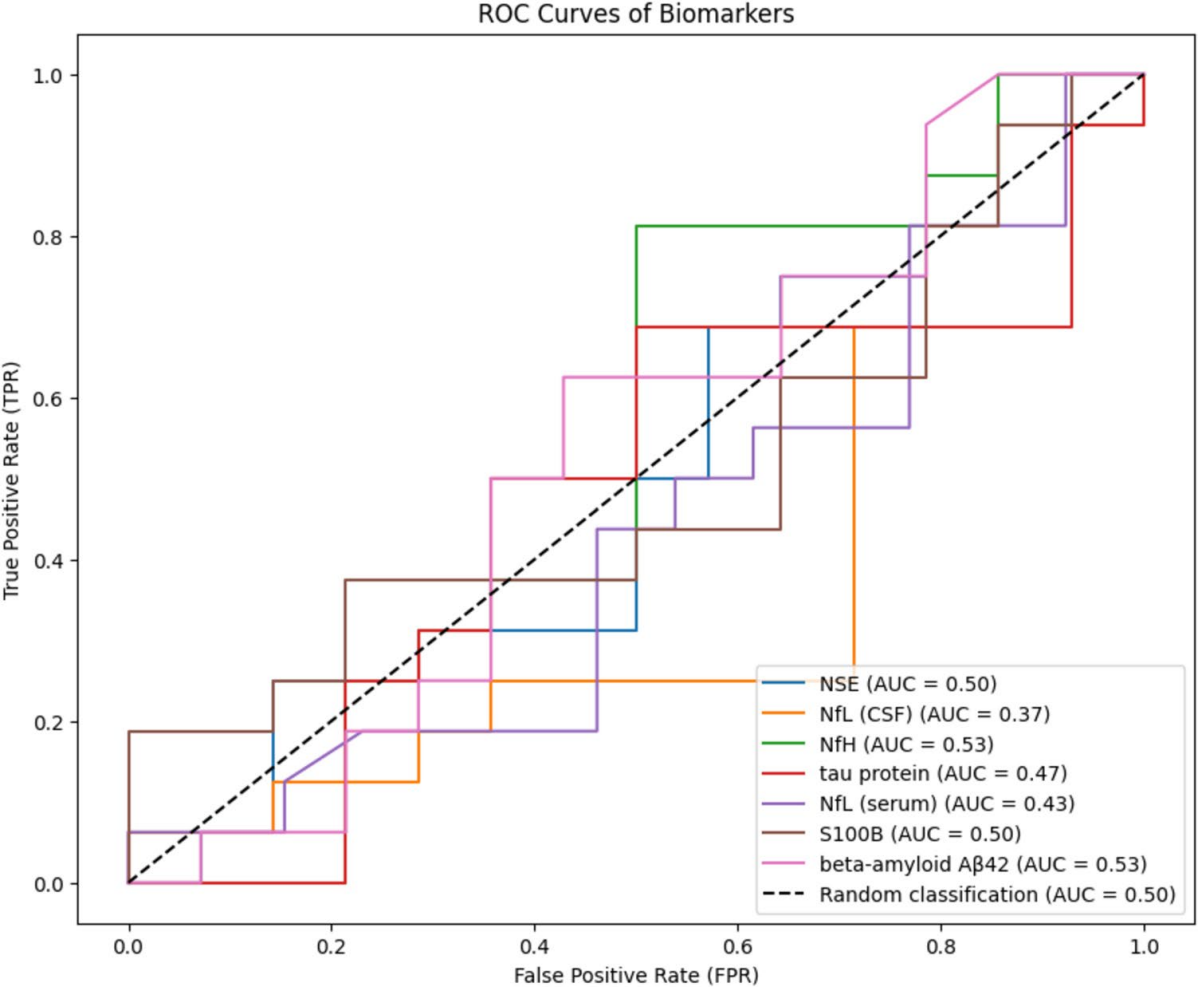
Graph of ROC analysis for each biomarker with calculation of AUC. NSE = Neuron-Specific Enolase; NfL = Neurofilament Light Chain; NfH = Neurofilament Heavy Chain; CSF = cerebrospinal fluid; AUC = area under curve

**Fig. 4:**
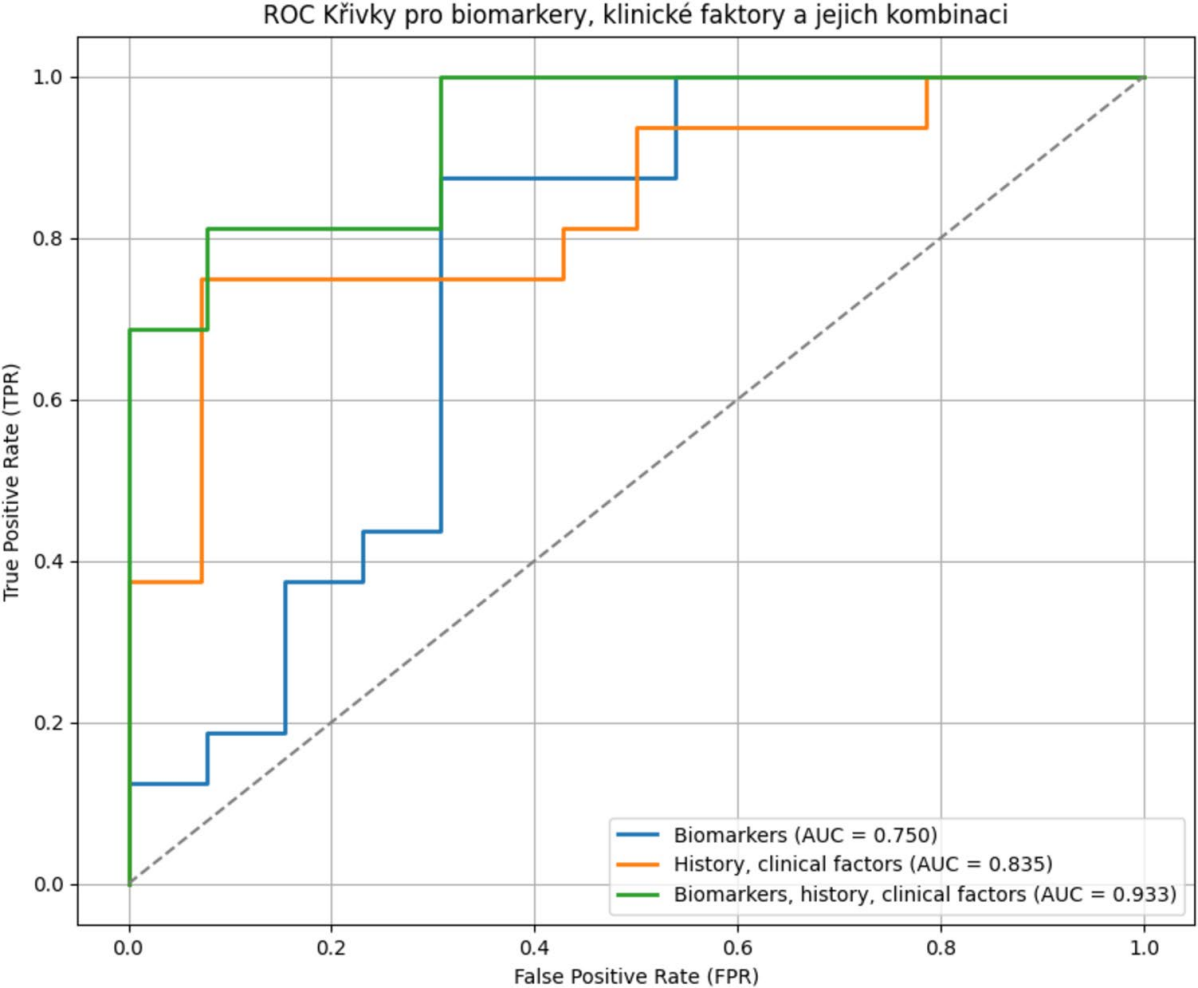
Graph of ROC analysis for predictive value all the examined biomarkers together, history and clinical factors and combination of history and clinical factors biomarkers with calculation of AUC. AUC = area under curve

No single biomarker demonstrated a superior predictive value (see Fig. 3). When logistic regression was applied, the area under the curve (AUC) for the combined biomarkers was 0.750. Consequently, the predictive value of patient history and clinical factors, particularly the iNPH scale, was evaluated. As shown in Fig. 4, incorporating clinical variables significantly enhanced model performance, yielding an AUC of 0.835, which further increased to 0.933 when combined with all seven examined biomarkers. The high AUC values obtained through logistic regression (up to 0.933) likely reflect overfitting, as evidenced by their sharp decline in cross-validation and Random Forest models.

However, subsequent analyses using cross-validation, and the Random Forest model revealed a substantial decline in predictive performance. The AUC dropped to 0.447 with cross- validation and 0.289 with the Random Forest model when biomarkers were analyzed together. For patient history and clinical factors alone, cross-validation and Random Forest yielded AUCs of 0.642 and 0.669, respectively. When biomarkers were combined with clinical factors, the AUC decreased to 0.428 with cross-validation and 0.431 with the Random Forest model.

## Discussion

In our study, no single biomarker demonstrated a strong predictive value for shunt responsiveness. We observed elevated levels of NfL and NfH in cerebrospinal fluid (CSF), along with a decreased concentration of beta-amyloid Aβ42.

However, in a more robust analysis using cross-validation and Random Forest models, the AUC dropped significantly, particularly for biomarkers and the combination of clinical history and biomarkers. All the seven examined biomarkers alone have poor predictive value for shunt responsiveness, with AUC values ranging from 0.289—0.447 in cross-validation and Random Forest models. In contrast, clinical factors are stronger predictors, achieving AUC values of 0.642—0.669, suggesting that patient history and clinical assessment (e.g., the iNPH scale) provide more reliable diagnostic information. Combining biomarkers with clinical factors did not enhance prediction; instead, it resulted in a decrease in AUC (0.428–0.431), indicating that biomarkers may introduce noise rather than contributing valuable information. The high AUC observed in logistic regression (0.750–0.933) on the full dataset was likely overestimated due to overfitting. Cross-validation and Random Forest models revealed the true generalizability of the predictions. The absence of significant correlations between biomarker levels and either patient age or symptom duration suggests that these markers may not directly reflect disease chronicity in iNPH, or that other confounding factors may obscure such relationships.

The limited predictive power of individual biomarkers may reflect their nonspecificity and biological variability. Many of these markers are elevated across a spectrum of neurodegenerative conditions and may not correlate well with dynamic clinical improvements following CSF removal. Furthermore, biomarker levels can be affected by sample handling, timing, and patient-specific pathology (e.g., concurrent Alzheimer’s disease), potentially masking true associations.

Clinical measures, particularly the iNPH scale and gait assessment, provide a real-time, functional evaluation of patient status and are more directly responsive to CSF pressure modulation. This may explain why they outperform static biomarkers that reflect underlying pathology but not its reversibility.

When comparing groups, no differences were detected. Although the NfL CSF/serum ratio was 1.75, no difference was found in our study. Importantly, all patients in Group A underwent VP shunt surgery and were systematically evaluated 3 months postoperatively. The INPH scale showed a statistically significant clinical improvement in all cases (median increase: 10.4 points, p = 0.00003). These results substantiate the validity of classifying Group A as true shunt responders based on postoperative clinical outcomes, not just preoperative testing. This provides a solid foundation for subsequent comparison of clinical and biomarker-based predictive models.

One limitation of the present study is that cerebrospinal fluid (CSF) samples were obtained exclusively via lumbar puncture. It is known that CSF composition may vary between the ventricular and lumbar compartments due to protein diffusion gradients. However, previous studies in iNPH suggest that while absolute biomarker concentrations can differ, relative trends and diagnostic value are generally preserved. This methodological consideration should be taken into account when interpreting biomarker levels.

Tullberg et al. (2007, 2008) [26, 27] demonstrated that CSF NfL levels decrease following shunt insertion in NPH patients, with the most significant reduction occurring within the first 6–12 months post-surgery. Serum NfL levels are also elevated in NPH patients [21]. Their studies further suggest that NfL dynamics could serve as a potential biomarker to distinguish responders from non-responders after shunt surgery [15, 21, 26–28]. Some authors have also highlighted its predictive value for shunt responsiveness [21, 32]. However, our study did not confirm these findings.

The relationship between NfH levels and hydrocephalus remains poorly understood, with only a limited number of studies available. The only study specifically addressing this topic was conducted by Tarnaris et al. [33], who found that NfH levels were negatively correlated with VEGF levels, suggesting an inverse relationship between neurodegeneration and angiogenesis/neurogenesis. However, they found NfH levels to be within the normal range. In contrast, our study identified elevated NfH levels in both responders and non-responders.

Our study found NSE levels to be within the normal range. This contrasts with the findings of Guzelcicek et al. [19], who reported significantly higher NSE levels in iNPH patients compared to healthy controls. Conversely, Mehmedika-Suljić et al. [21] reported the opposite results, showing no significant increase in NSE levels.

Some studies suggest that tau protein levels may predict certain clinical aspects in iNPH patients. Lower tau-protein levels have been associated with a long-lasting response to VP shunting, whereas higher tau-protein levels were linked to only temporary postoperative improvement with rapid early decline [29]. However, elevated tau-protein levels did not preclude a positive surgical outcome. Typically, iNPH patients exhibit normal tau-protein levels [34]. Furthermore, the presence of Alzheimer’s disease and elevated tau-protein levels were not considered contraindications for VP shunting [34]. Despite these findings, tau protein alone is not recommended as a sole diagnostic or prognostic biomarker.

The role of S100B in shunt responsivity has not been clarified, yet. The insights from its role in other neurological disorders suggest that S100B may influence the outcomes of shunt surgery through its modulation of neuroinflammation, oxidative stress, and astrocyte activation. Further research is needed to explore the specific role of S100B in the context of NPH and shunt responsivity, as well as its potential utility as a biomarker for predicting surgical outcomes [15, 16, 35].

Beta-amyloid Aβ42, particularly its toxic conformers, might plays a role in predicting shunt responsivity in NPH [15]. However, its utility is enhanced when considered alongside other biomarkers and clinical factors. The relation between Aβ42 and Alzheimer’s disease pathology in NPH underscores the complexity of shunt outcomes [15, 23].

While several previous studies (e.g., Tullberg et al., Ågren-Wilsson et al., Pfanner et al.) have suggested that biomarkers such as NfL or tau protein may have predictive value for shunt responsiveness, our findings did not confirm these results [26–28, 32]. This discrepancy may stem from differences in patient selection, sampling timing, biomarker quantification methods, or follow-up duration. Our results underscore the need for standardized protocols and larger prospective cohorts.

Our study has several limitations. The primary is the relatively small sample size, which was determined based on a power analysis as part of a pilot study design. A post hoc power analysis revealed that the statistical power for detecting significant differences across biomarkers was substantially lower than the conventional threshold of 0.8, ranging from 0.05 to 0.23. The highest observed power (0.234) was for NfL in CSF, whereas all other biomarkers exhibited lower power, suggesting a high likelihood of Type II errors (false negatives). This discrepancy reflects the difference between the expected and observed effect sizes. Although we initially planned for 36 patients to account for potential dropouts, the final cohort of 30 patients may have further reduced statistical sensitivity. Another limitation is that we did not perform confirmatory Western blot analysis to identify specific isoforms of Tau, Amyloid-β, or S100B proteins. As the ELISA kits used may detect multiple isoforms, we cannot fully distinguish whether the measured signal corresponds to a single molecular species or a combination. Future studies incorporating Western blot or mass spectrometry could help clarify this issue.

Furthermore, while postoperative outcome data were rigorously evaluated and confirmed in all patients who underwent surgery (Group A), patients in Group B did not undergo shunt placement. As such, their true responsiveness remains unknown. This limits our ability to detect false negatives—patients who may have been misclassified as non-responders but could have benefited from surgery. Future studies incorporating long-term observational follow-up or ethically designed crossover protocols may help address this limitation.

Beyond sample size constraints, potential confounding factors, including comorbidities and medication effects, may have influenced biomarker levels and clinical outcomes, adding complexity to the interpretation of our findings. These factors highlight the need for larger, well-controlled studies to confirm our results and reduce the risk of bias.

## Conclusion

No single biomarker demonstrated a strong predictive value for shunt responsiveness. Biomarkers alone have poor predictive value for shunt responsiveness. In contrast, clinical factors are stronger predictors, suggesting that patient history and clinical assessment (e.g., the iNPH scale) provide more reliable diagnostic information. Combining biomarkers with clinical factors did not enhance prediction.

## Data Availability

No datasets were generated or analysed during the current study.
